# Numerical Modeling of the Effect of Cutting-Edge Radius on Cutting Force and Stress Concentration during Machining

**DOI:** 10.3390/mi13020211

**Published:** 2022-01-28

**Authors:** Peng Li, Zhiyong Chang

**Affiliations:** 1 Department of Mechanical Engineering, Northwestern Polytechnical University, Xi’an 710072, China; lip973@mail.nwpu.edu.cn; 2 Institute for Aero-Engine Smart Assembly of Shaanxi Province, Xi’an 710072, China

**Keywords:** cutting-edge radius, shear angle, cutting force, stress concentration depth, finite element analysis

## Abstract

Cutting is the primary method of material removal, and the quality of machined parts depends on the geometry of cutting tools. In this paper, a new cutting force coefficient model is established, revealing the influence of cutting-edge radius on the cutting process. The effects of cutting-edge radius on the shear angle and cutting force components are analyzed by finite element simulations. A series of simulations is conducted, and the results show that with increased cutting-edge radius, the shear angle decreases nonlinearly, and the cutting force increases gradually. Additionally, the growth rate of the feed force caused by increasing the cutting-edge radius is higher than that of the tangential force. Furthermore, the stress concentration area of the machined surface extends from the surface to the subsurface as the cutting-edge radius increases. The results of this research show that changing the cutting edge affects the cutting force component, shear angle, and stress concentration range during the cutting process. These results provide a theoretical reference for predicting the residual stress in parts.

## 1. Introduction

Cutting machining is a common manufacturing method that is widely used in industrial applications, such as the precision machining of optical parts. Cutting-edge radius (CER) affects the cutting force [1], cutting temperature [2], and surface integrity of parts [3]. Therefore, many scholars are engaged in cutting force analysis and modeling [4,5,6,7,8,9].

Cutting force is one of the critical factors during the cutting process and is closely related to the cutting parameters. Vijayaraghavan et al. [4] used an artificial neural network to analyze the cutting force with cutting depth and feed rate. Zhuang [5] established a cutting force model of a chamfered edge and found that cutting force increased nonlinearly with the chamfering edge’s length. Davis et al. [6] experimentally studied the changes in shear strain and strain rate with cutting speed and analyzed the strain rate distribution in chip flow direction. However, the above research [4,5,6] explored only the magnitude of cutting force and did not profoundly reveal the reasons for its change. In addition, some scholars analyzed the effect of cutting force based on elastic–plastic interactions. Chen et al. [7] established a theoretical model considering the elastic, plastic, and brittle zones. Error factors are inevitable during the cutting process, Wojciechowski et al. [8] developed a milling force model that considers the geometry errors of machining system. Wu et al. [9] found that serrated chips caused cutting force variations. Wojciechowski and Mrozek [10] minimized cutting force by optimizing the inclination angle of the tool axis.

Unfortunately, the research of [4,5,6,7] ignored the influence of CER. During the early development of cutting processes, it was assumed that the cutting edge was sharply acceptable for large-scale cutting because the CER was much smaller than the undeformed chip thickness [11]. However, in micron-scale cutting, the undeformed chip thickness is close to the CER, and the CER is a vital parameter that cannot be ignored. Fang et al. [12] proposed the relationship between the effective rake angle and the CER. With the rapid development of computer technology, Woon et al. [13,14] used the finite element method to study the relationship between chip formation and CER. The CER was constant along the tool corner [13,14]; however, Ozel [15] designed a tool in which the CER changed, and used the finite element simulation to analyze its cutting performance. Different from the finite element method, Amir et al. [16] predicted cutting force with smooth particle hydrodynamics. In [11,12,13], the effects of the CER on cutting force and cutting temperature were investigated, but the studies did not reveal the evolution of involved parameters in the cutting force. Therefore, some scholars began to explore the influence of CER on parameters [17,18,19].

Rake angle is an important parameter of a tool, and some scholars are engaged in the effects of CER on the actual working rake angle of cutting tools. Lai et al. [17] considered that CER changes the rake angle of the tool, and the undeformed chip thickness is directly proportional to the CER. Yang et al. [18] established a two-dimensional mathematical model of the cutting force that considers the CER. Later, Dai et al. [19] proposed a method based on the CER to evaluate the cutting force coefficient. Aramcharoen and Mativenga [20] found that when the uncut chip thickness is equal to the CER, the best surface finish can be obtained; they suggest that the CER can greatly improve the burr on parts. With the development of cutting technology, ultrasonic-assisted cutting was used, and it was observed that the undeformed chip thickness increased with the CER [21].

The experimental research cost on cutting force is high, and unique fixtures need to be designed for small parts, which is cumbersome and laborious. Therefore, this paper proposes a finite element analysis model to analyze the evolution of the shear angle and explore the variational principle of cutting force components with CER; thus, it is not the magnitude of cutting force and stress of existing theories, but a new insight of evolution. In addition, the evolution of stress propagation is characterized first by the depth of stress concentration. The research results are relevant to the selection of cutting tools and optimization of tool design. Finally, this study provides a new theoretical reference for controlling the surface integrity of parts.

## 2. Simulation Modeling

Traditional cutting tools are assumed to be perfectly sharp, although they undergo wear quickly during the cutting process. With the upgrading of tool-manufacturing technology and the demand for energy saving and carbon emissions reduction, it is crucial to improve the lifespan of tools. For this reason, it is becoming common to use tools with a CER that can greatly increase tool strength, especially for difficult-to-machine materials. The shear angle is an important physical parameter that affects the cutting force. This section establishes a cutting force model and gives the relationship between the cutting force and shear angle.

### 2.1. Tool Geometry and Mechanical Model

**Figure 1:**
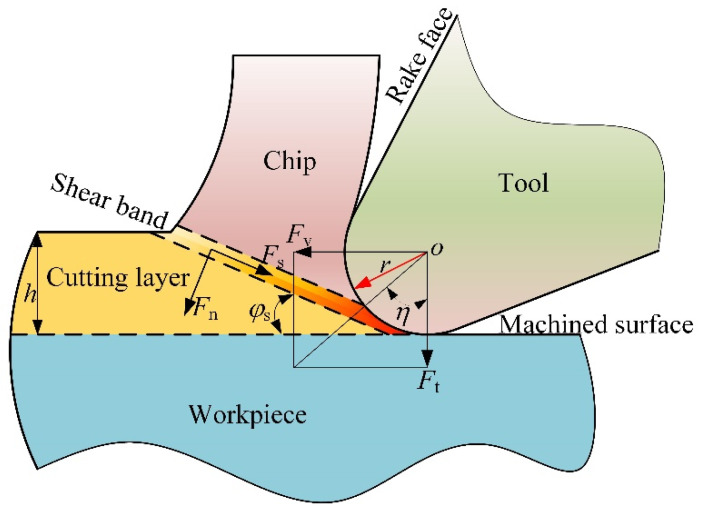
Geometry of the tool and workpiece simulation model.

Material undergoes large plastic deformation during cutting, and chips form when the cutting layer undergoes shear deformation. Chips move along the rake face of the tool after shear deformation, as shown in Figure 1, where $F_{v}$ and $F_{t}$ are the tangential force and the feed force, respectively, $F_{v}$ is in the direction of velocity, and $F_{t}$ is in the direction of uncut chip thickness. In the shear plane are normal force $F_{s}$ and shear force $F_{n}$, respectively. The acute angle formed by $F_{v}$ and $F_{t}$ is η; r is the CER; $\varphi_{s}$ is the shear angle; and h is the uncut chip thickness.

According to [22], the shear zone can be assumed to be a thin plane, and the shear band thickness can be approximated as zero. Therefore, the normal stress $\sigma_{s}$ and the shear stress $\tau_{s}$ in the shear plane can be expressed as:

(1) $$\begin{array}{l} \tau_{w}=\frac{F_{s}}{A_{s}}\\ \sigma_{w}=\frac{F_{n}}{A_{s}} \end{array}$$

where $A_{s}$ is the shear plane area.

According to [23], the tool–workpiece intersection forces $F_{v}$ and $F_{t}$ in orthogonal cutting can be expressed as

(2) $$\begin{array}{l} F_{v}=bh\left(\tau_{w}\frac{\cos\left(\beta_{f}-\beta_{r}\right)}{\sin\left(\varphi_{s}\right)\cos\left(\varphi_{s}+\beta_{f}-\beta_{r}\right)}\right)\\ F_{t}=bh\left(\tau_{w}\frac{\sin\left(\beta_{f}-\beta_{r}\right)}{\sin\left(\varphi_{s}\right)\cos\left(\varphi_{s}+\beta_{f}-\beta_{r}\right)}\right) \end{array}$$

where $\beta_{f}$ and $\beta_{r}$ are the friction angle and rake angle, respectively. The friction angle is affected by the state of the contact between the tool and the workpiece, rake face roughness, and lubrication by the cutting fluid during the cutting process.

Based on the maximum shear stress principle, the shear angle $\varphi_{s}$, the friction angle $\beta_{f}$, and the rake angle $\beta_{r}$ can be expressed as:

(3) $$\varphi_{s}+\beta_{f}-\beta_{r}=\frac{\pi }{4}$$

According to [22], the average friction coefficient is assumed to be constant for a given tool–workpiece material. Substituting (3) into (2) then simplifies to

(4) Fv=2bh(τwcos(π4−φs)sin(φs))Ft=2bh(τwsin(π4−φs)sin(φs))

To characterize the effect of the shear angle on the cutting force, Equation (4) is written as

(5) $$\begin{array}{l} F_{v}=\sqrt{2}bh\tau_{w}k_{v}\left(\varphi_{s}\right)\\ F_{t}=\sqrt{2}bh\tau_{w}k_{t}\left(\varphi_{s}\right) \end{array}$$

where $k_{v}\left(\varphi_{s}\right)$ and $k_{t}\left(\varphi_{s}\right)$ are the tangential force coefficient and the feed force coefficient, respectively. The cutting force coefficient can be written as

(6) kv(φs)=cos(π4−φs)sin(φs)kt(φs)=sin(π4−φs)sin(φs)

The cutting force coefficient includes the shear yield according to [22]. However, in this work, the cutting force coefficients $k_{v}\left(\varphi_{s}\right)$ and $k_{t}\left(\varphi_{s}\right)$ are different from those in [22]. Instead, $k_{v}\left(\varphi_{s}\right)$ and $k_{t}\left(\varphi_{s}\right)$ are functions of the shear angle $\varphi_{s}$ and are nondimensional in the present model.

**Figure 2:**
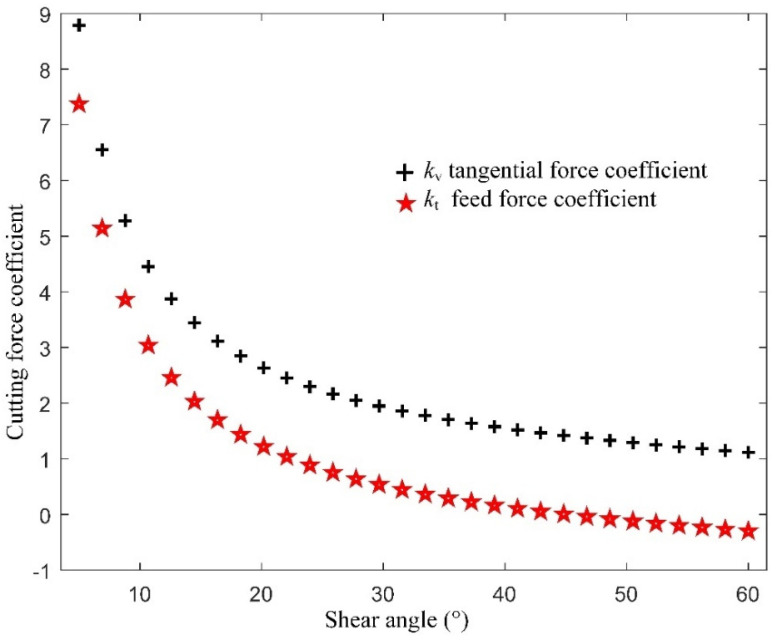
Cutting forces with different shear angle.

To understand the effect of the shear angle on cutting force coefficients $k_{v}\left(\varphi_{s}\right)$ and $k_{t}\left(\varphi_{s}\right)$, a sensitivity study was conducted by changing the $\varphi_{s}$, and calculating $k_{v}\left(\varphi_{s}\right)$ and $k_{t}\left(\varphi_{s}\right)$ based on Equation (6). The result is shown in Figure 2.

Figure 2 shows that as the shear angle increases, the cutting force coefficients $k_{v}\left(\varphi_{s}\right)$ and $k_{t}\left(\varphi_{s}\right)$ decrease nonlinearly, which means that the material deforms more easily as the shear angle increases. The tangential force coefficient $k_{v}\left(\varphi_{s}\right)$ is always larger than the feed force coefficient $k_{t}\left(\varphi_{s}\right)$ at the same shear angle.

The acute angle η between the tangential cutting force $F_{v}$ and feed force $F_{t}$ can be expressed as

(7) $$\eta =\arctan\left(\frac{F_{v}}{F_{t}}\right)$$

where η reflects the direction change in the resultant cutting force.

### 2.2. Workpiece Property

The Johnson–Cook constitutive model is extensively utilized in metal-cutting modeling because it can adequately describe the behavior of a workpiece undergoing large strains, thermal softening, and high strain rate.

(8) $$\sigma =\left(A+B\varepsilon^{n}\right)\left(1+C\mathrm{In}\left(1+\frac{\overset{\cdot }{\varepsilon }}{\varepsilon_{0}}\right)\right)\left(1-{\left(\frac{T-T_{r}}{T_{m}-T_{r}}\right)}^{m}\right)$$

where $\sigma ,\varepsilon^{n},\overset{\cdot }{\varepsilon },\;\mathrm{and}\;\varepsilon_{0}$ are the flow stress, plastic strain, plastic strain rate, and equivalent plastic strain, respectively; *T*, *T_r_*, and *T_m_* are the current material temperature, melting point, and room temperature, respectively. *A*, *B*, *C*, *m*, and *n* are constitutive constants: *A* is the initial yield of materials; *B* is the stress hardening modulus; *C* is the sensitivity coefficient; *m* is the thermal coefficient of softening; and *n* refers to the hardening index. The flow behavior of GH 4169 is governed by the Johnson–Cook model. The *A* = 1241 Mpa, *B* = 622 Mpa, *C* = 0.0134, *m* = 1.3, and *n* = 0.652 are the parameters for GH 4169 as given by [24]. To investigate the CER on shear angle, cutting force, and stress concentrate, the values of the CER used in this simulation are shown in Table 1. The rake angle of the tool was 5°, the relief angle was 5°. The cutting speed was 30 m/min, and the feed rate was set to 0.15 mm. The tool material was cemented carbide, and the cutting tool was not coated.

GH 4169 is a nickel-based superalloy widely used in the aerospace industry. However, fast tool wear and high cutting temperature make cutting this material challenging. The composition of GH 4169 is presented in Table 2.

The physical properties of GH 4169 are presented in Table 3. The cutting tool is assumed to be perfectly rigid solids. The workpiece GH 4169 is a rectangular block with 2 mm length, 0.5 mm width, and 1 mm height.

## 3. Results and Discussion

The cutting process is complex and involves high temperature, high pressure, and large strain. The force and stress on the tool–workpiece system are important factors that determine the energy required. The shear angle is the critical variable used to analyze the cutting force; therefore, the shear angle is investigated first in this study. This section studies the effects of CER on shear angle, cutting force, and stress concentration depth. A series of orthogonal simulations with the finite element method software AdventEdge are conducted.

### 3.1. Evolution of Shear Angle

The acute angle between the shear plane and the cutting speed direction is called the shear angle. The shear angle affects cutting force consumed by cutting, and a large shear angle saves energy. According to Equation (5), the cutting force is a function of the shear angle. Therefore, it is helpful to analyze the influence of the CER on the shear angle to deeply reveal the mechanism of the effect of the CER on the cutting force.

**Figure 3:**
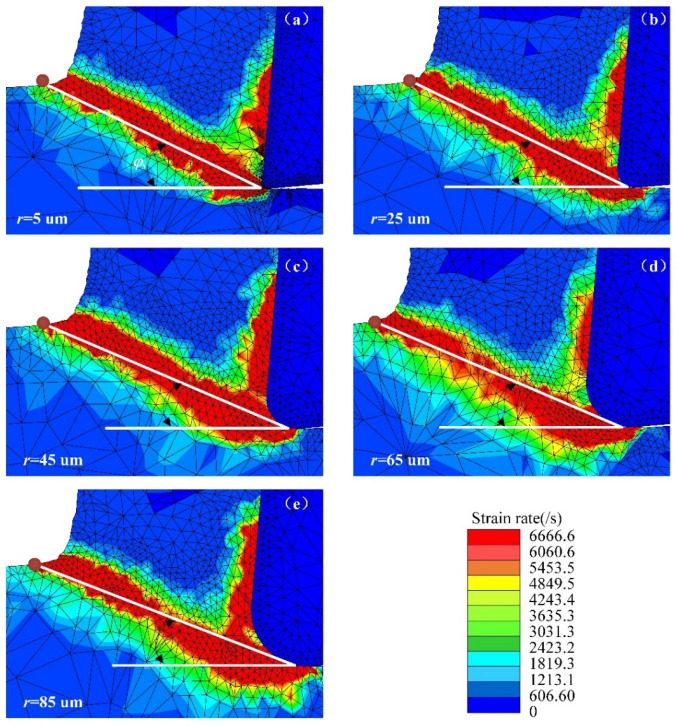
Bending point position versus cutting-edge radius; (**a**) *r* = 5 µm; (**b**) *r* = 25 µm; (**c**) *r* = 45 µm; (**d**) *r* = 65 µm; (**e**) *r* = 85 µm.

Figure 3 shows the results of the cutting simulation with different CERs, and measurement of the shear angle. Figure 3a–e shows that the cutting layer material deforms in the shear plane, so the strain rate in the shear plane is very high. As the CER increases, the position of the chip bending point shifts, resulting in a change in the shear angle.

**Figure 4:**
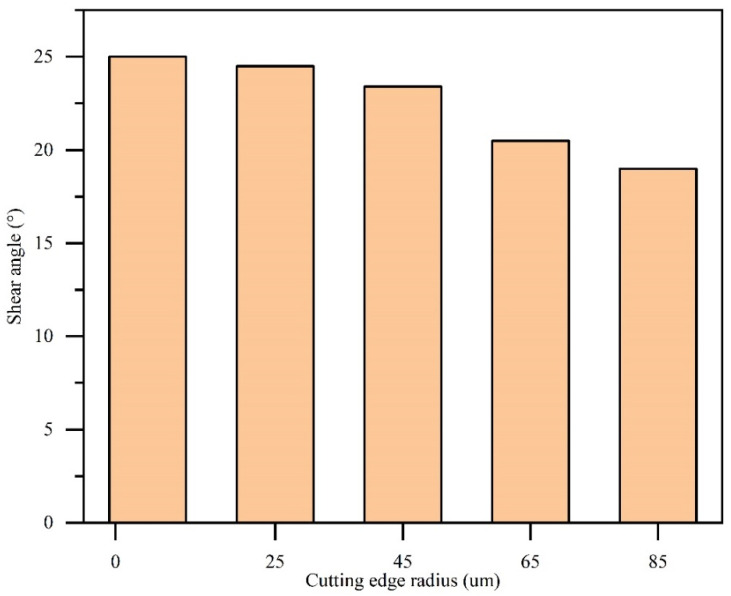
Shear angle for various cutting-edge radius.

The shear angle was measured in each simulation, as shown in Figure 4. When the CER is 5 µm, the shear angle is 25°; as the CER increases to 85 µm, the shear angle decreases to 19°, a reduction of by 24%. Increasing the CER reduces the shear angle, since increasing the CER decreases the average rake angle of the tool. According to Equation (3), the shear angle decreases as the rake angle decreases.

### 3.2. Von Mises Stress of the Machined Surface

The tool–workpiece system undergoes contact stress during the cutting process, and the material forms a chip after large plastic deformation. The local area inside the workpiece where the stress is significantly higher than the surrounding region is called the stress concentration.

**Figure 5:**
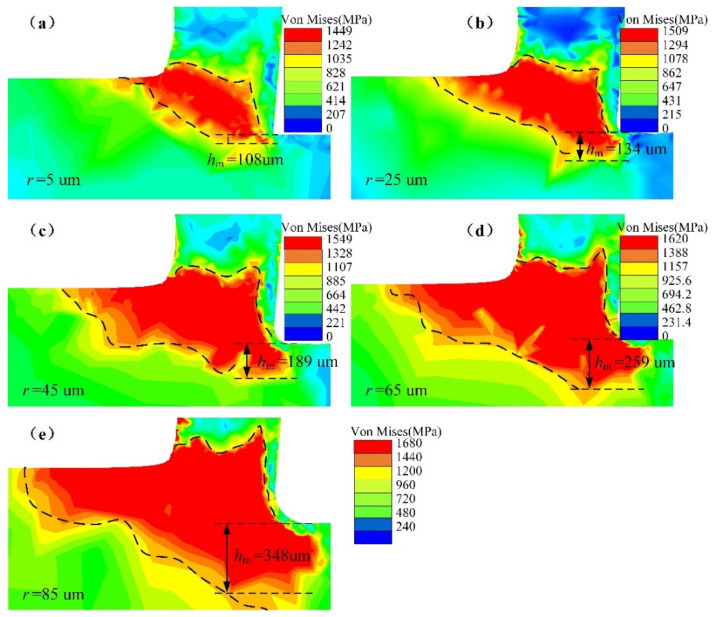
Von Mises stress distribution on a workpiece; (**a**) *r* = 5 µm; (**b**) *r* = 25 µm; (**c**) *r* = 45 µm; (**d**) *r* = 65 µm; (**e**) *r* = 85 µm.

Figure 5a–e shows the von Mises stress distribution of the workpiece. The region of stress concentration expanded with increasing CER. In Figure 5a, the stress concentration area is mainly on both sides of the shear band due to shear deformation. However, in Figure 5e, the stress concentration area extends from the shear band to the machined surface and cutting layer, and the region of the workpiece stress concentration expands with CER. The results indicate that the CER induces stress to expand toward the cutting layer and the workpiece surface. However, the stress concentration does not extend to the chip, mainly because the cutting layer undergoes upward flow and concave bending after shear deformation, the chip surface is free, and the chip material can flow uniformly. Because the machined part is a fixed constraint during the cutting process, the stress propagates and expands to the cutting layer and the machined surface.

Although the stress expands to the machined layer, the cutting layer is removed in the next cycle. The machined surface affects the service life of the part. The depth of stress concentration is measured from the machined surface along the direction of undeformed chip thickness. The depth of stress concentration affects the fatigue life of the part and makes an important contribution to the residual stress of the surface and subsurface. The depth of stress concentration is measured to characterize the degree of stress concentration, as shown in Figure 5. The stress depth $h_{m}$ is 108 µm in Figure 5a, while the stress concentration depth $h_{m}$ is 348 µm in Figure 5e, and the depth of the stress concentration is 3.2 times when *r* = 85 µm than when *r* = 5 µm. As shown in Figure 5a–e, the depth of stress concentration gradually increases with increasing CER.

**Figure 6:**
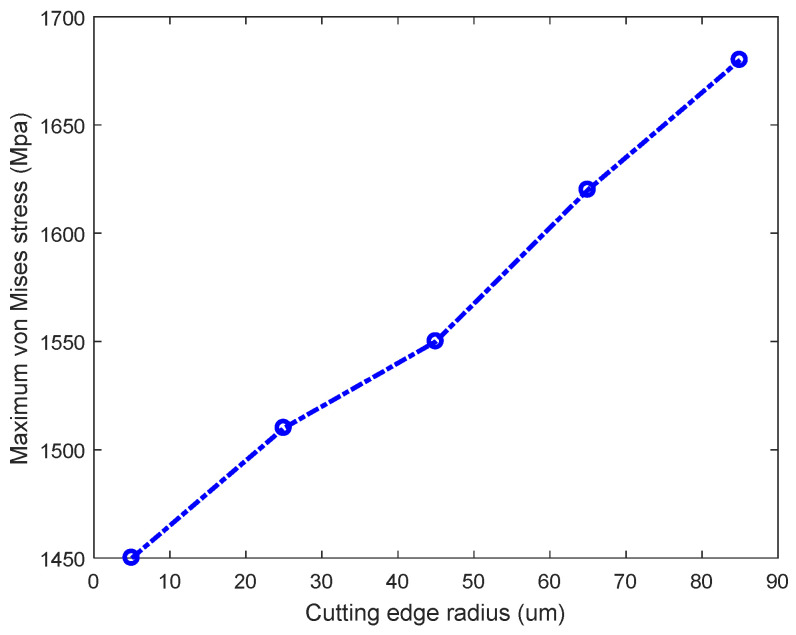
Maximum von Mises stress on the workpiece.

Figure 6 shows the maximum von Mises stress variation for different CERs. When the CER is 5 µm, the maximum stress is 1450 MPa. The maximum stress is 1680 MPa with *r* = 85 µm; the ultimate stress increases by 230 MPa compared with *r* = 5 µm. Thus, increasing the CER increases the amplitude of the maximum stress. The average rake angle of the CER is less than the design rake angle of the tool, increasing the maximum stress.

An increase in the stress concentration depth changes the residual stress of the surface and subsurface of the workpiece, increases the final residual stress of the part, and affects the fatigue of the part. Increasing the CER expands the stress concentration depth and amplitude. Additionally, the expansion of the stress concentration range increases energy consumption. These simulation results will provide a reference for efforts to control the residual stress of parts.

### 3.3. Effect of the CER on Cutting Forces

Cutting force is important for analyzing and controlling workpiece deformation. The influence of the CER on the cutting forces is neglected in the traditional cutting process. This section uses simulations to explore the effects of the CER on the cutting force.

**Figure 7:**
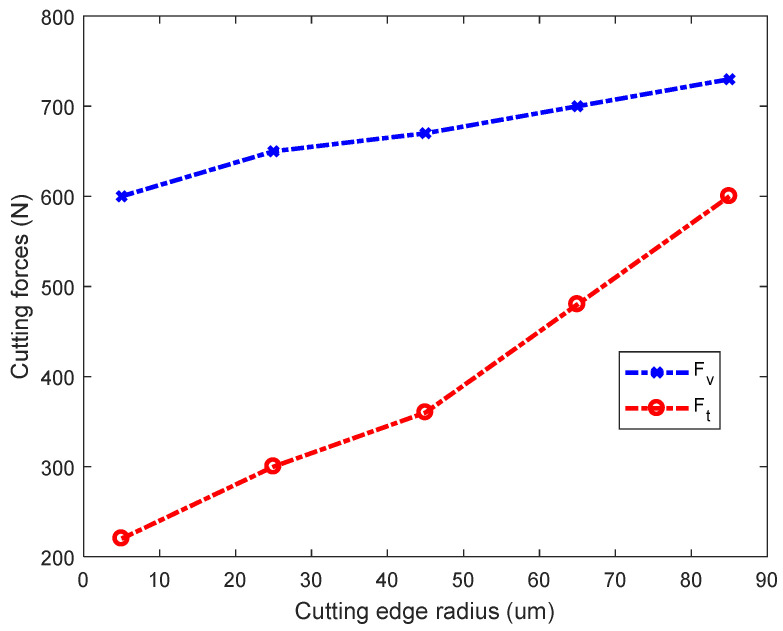
Cutting force for different cutting-edge radii.

As shown in Figure 7, the tangential force $F_{v}$ and the feed force $F_{t}$ are 600 N and 220 N in Test No.1, respectively; they increase to 730 N and 600 N in Test No.5, respectively. The tangential force and feed force rise by 22% and 173%, respectively. Therefore, the cutting force and feed force increase with the CER, mainly because the shear angle decreases as the CER increases, as shown in Section 3.1; thus, the cutting force increases. These simulation results are consistent with the theoretical analysis of Equation (5). In every case, the tangential force $F_{v}$ is greater than the feed force $F_{t}$ in Figure 7. The main cutting force is the tangential force during the cutting process, which determines the power required. However, the feed force $F_{t}$ has a much faster growth rate than the tangential force $F_{v}$, which indicates that the CER mainly affects the feed force. Due to the vibration and deformation of the workpiece, cutting inevitably produces friction on the machined surface. The CER increases the contact area between the flank and the machined surface, so the normal pressure between the tool and the machined surface also increases.

**Figure 8:**
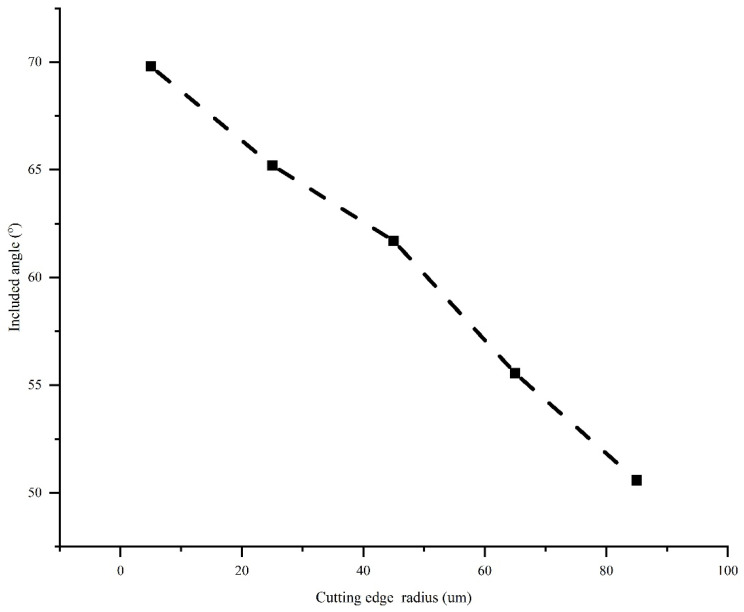
The included angle between the tangential force and the feed force.

The angle η between the tangential force and the feed force is shown in Figure 8. The angle η decreases with the CER mainly due to the quick increase in feed force as the CER. Additionally, increasing the CER causes the transition from the rake face to the flank face to no longer be a simple straight surface, but rather a complex one. Contact with the workpiece is also a complex surface condition. A decrease in the angle η means that the direction of the resultant force changes.

## 4. Conclusions

Force is one of the crucial factors related to tool life and the quality of machined parts. CER is the key parameter of the geometry of the cutting tool that cannot be ignored in micromachining. This paper proposed a new method for the calculation of the cutting force coefficient based on an expression for the coefficient as a nonlinear function of the shear angle. The effect of CER on the shear angle was analyzed by simulations, and its influence on the cutting force was further studied. The main conclusions were as follows:

1. The cutting force increased with the CER. The simulation analysis showed that the cutting force coefficient decreased as the shear angle increased, which was consistent with the analytical cutting force model. As the CER increased, the shear angle decreased, which caused the cutting force to increase.
2. The increase in the feed force was quicker than the increase in the tangential force with increasing CER. With enhanced CER, the rate of feed force growth was much higher than that of the tangential force, unlike the behavior of traditional sharp cutter. The effect of the CER on the feed force must be considered in order to understand tool life and the quality of machined parts.
3. The stress concentration range of the workpiece gradually expanded with increasing CER. Simulation experiments showed that the stress concentration area extended from the shear band to the cutting layer and the subsurface of the workpiece, resulting in a substantial increase in the stress concentration depth, which affected the surface and integrity of the workpiece. Additionally, the maximum stress in the workpiece increased with the CER.

## Figures and Tables

**Table 1:** Cutting-edge radius used in this simulation.

Test No.	1	2	3	4	5
Cutting-edge radius *r* (µm)	5	25	45	65	85

**Table 2:** Chemical composition of GH4169 [25].

Element	Ni	Cr	Nb	Mo	Ti	Al	Co	Mn	Cu	Si	C	Fe
Wt %	52.15	19.26	5.03	3.03	1.08	0.56	0.5	0.22	0.1	0.26	0.052	17.75

**Table 3:** Typical mechanical properties at room temperature [25].

Tensile Strength (MPa)	Yield Strength (MPa)	Young’s Modulus (GPa)	Density (g∙cm^−3^)	Poisson’s Ratio	Thermal Conductivity (W/m∙K)
1430	1300	204	8.24	0.3	14.7
